# Reconstruction options following pancreaticoduodenectomy after Roux-en-Y gastric bypass: a systematic review

**DOI:** 10.1186/s12957-018-1467-6

**Published:** 2018-08-13

**Authors:** William F. Morano, Mohammad F. Shaikh, Elizabeth M. Gleeson, Alvaro Galvez, Marian Khalili, John Lieb, Elizabeth P. Renza-Stingone, Wilbur B. Bowne

**Affiliations:** 1 Division of Surgical Oncology, Department of Surgery, Drexel University College of Medicine, 245 N. 15th Street, Suite 7150, Philadelphia, PA 19102 USA; 2 Division of Minimally Invasive Surgery, Department of Surgery, Drexel University College of Medicine, 245 N. 15th St, Suite 7150, Philadelphia, PA 19102 USA; 3 Division of Gastroenterology & Hepatology, Department of Medicine, Drexel University College of Medicine, 219 N Broad St, 5th Floor, Philadelphia, PA 19107 USA

**Keywords:** Roux-en-Y gastric bypass, Pancreaticoduodenectomy, Whipple, Pancreatic cancer, Bariatrics

## Abstract

**Background:**

Obesity is a risk factor for pancreatic cancer which may be treated with Roux-en-Y gastric bypass and represents an increasing morbidity. Post-RYGB anatomy poses considerable challenges for reconstruction after pancreaticoduodenectomy (PD), a growing problem encountered by surgeons. We characterize specific strategies used for post-PD reconstruction in the RYGB patient.

**Methods:**

PubMed search was performed using MeSH terms “Gastric Bypass” and “Pancreaticoduodenectomy” between 2000 and 2018. Articles reporting cases of pancreaticoduodenectomy in post-RYGB patients were included and systematically reviewed for this study.

**Results:**

Three case reports and five case series (25 patients) addressed PD after RYGB; we report one additional case. The typical post-gastric bypass PD patient is a woman in the sixth decade of life, presenting most commonly with pain (69.2%) and/or jaundice (53.8%), median 5 years after RYGB. Five post-PD reconstructive options are reported. Among these, the gastric remnant was resected in 18 cases (69.2%), with reconstruction of biliopancreatic drainage most commonly achieved using the distal jejunal segment of the pre-existing biliopancreatic limb (73.1%). Similarly, in the eight cases where the gastric remnant was spared (30.8%), drainage was most commonly performed using the distal jejunal segment of the biliopancreatic limb (50%). Among the 17 cases reporting follow-up data, median was 27 months.

**Conclusion:**

Reconstruction options after PD in the post-RYGB patient focus on resection or preservation gastric remnant, as well as creation of new biliopancreatic limb. Insufficient data exists to make recommendations regarding the optimal reconstruction option, yet surgeons must prepare for the possible clinical challenge. PD reconstruction post-RYGB requires evaluation through prospective studies.

## Background

Morbid obesity, a known risk factor for the development of pancreatic cancer, may be treated surgically with Roux-en-Y gastric bypass (RYGB). The post-bypass anatomy can make reconstruction after pancreaticoduodenectomy more complex, with multiple surgical options. Although uncommon, this situation will be encountered more frequently as the post-RYGB population increases in size. Few reported cases exist to provide evidence-based guidelines for options for reconstruction of the post-pancreaticoduodenectomy anatomy in a patient with prior Roux-en-Y gastric bypass.

## Introduction

Obesity is a growing problem in the USA and known risk factor for development of pancreatic malignancy [1–3]. The Roux-en-Y gastric bypass (RYGB) has proven to be an effective, long-term solution for obesity and its associated morbidities [4–6]. RYGB addresses the problem of obesity in two ways: a restrictive component involving the creation of a gastric pouch with alimentary limb, and a malabsorptive component bypassing the proximal portion of the small intestine. The resultant configuration is a significant reconstruction and poses potential future diagnostic and therapeutic challenges [7–11]. In the case of pancreatic malignancy requiring pancreaticoduodenectomy (PD), the surgeon must consider whether or not to resect the gastric remnant, as well as the method of reconstruction of the biliopancreatic and alimentary limbs [12]. To date, few case reports or series of post-RYGB pancreaticoduodenectomies have directly addressed this challenging clinical scenario. With the growing prevalence of obese patients undergoing RYGB, surgeons will be facing similar issues more frequently in the future. We present such a case and systematically review the existing literature to report management strategies and discuss relevant considerations for reconstruction.

## Methods

**Fig. 1:**
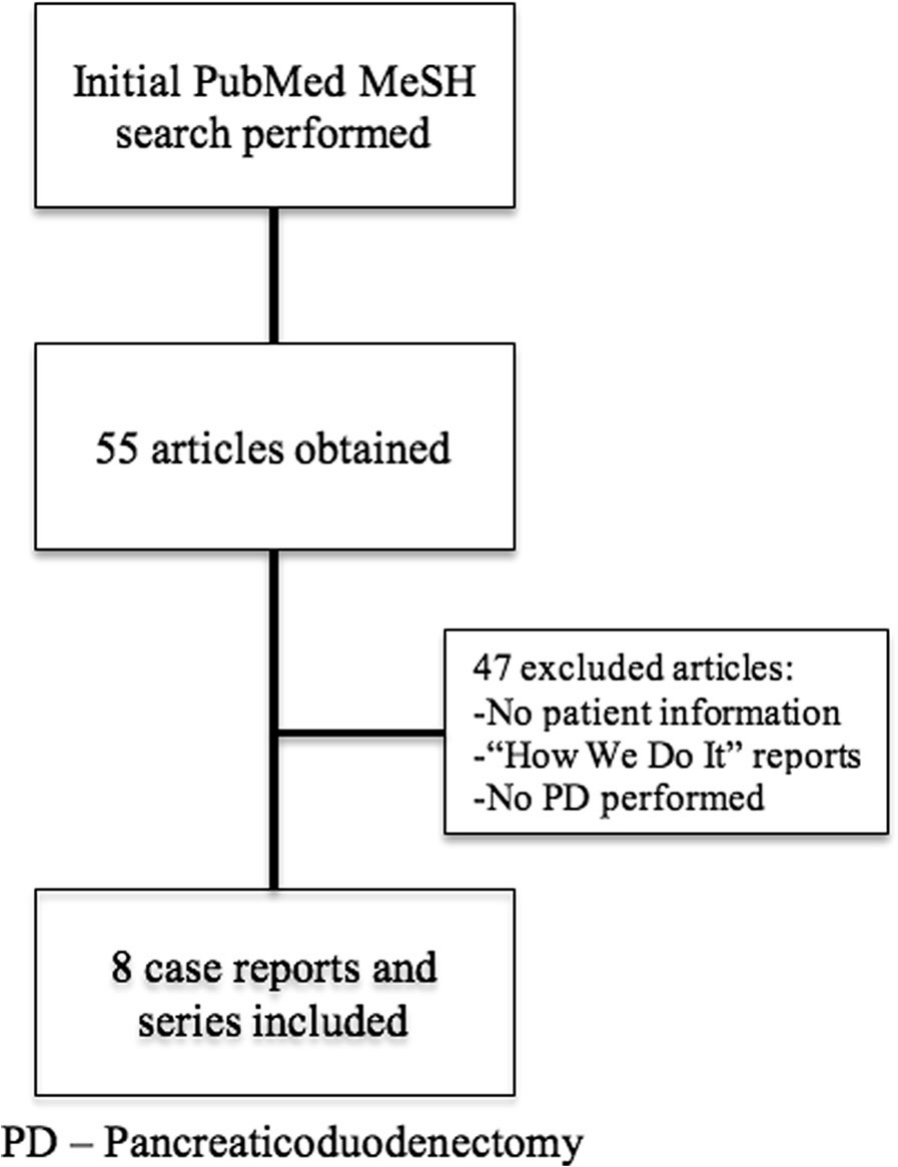
Flow chart depicting literature search and criteria for exclusion for final review

PubMed search was performed using MeSH terms “Gastric Bypass” and “Pancreaticoduodenectomy” between 2000 and 2018. We systematically reviewed and extracted data from included cases such as patient-related demographics, diagnosis, operative techniques, and outcomes. Articles in which no patient data was provided, operative technique-specific, and reports in which PD was not performed after RYGB were excluded from this review (Fig. 1). Qualitative variables are reported as proportions. Continuous quantitative variables are provided as medians with interquartile ranges.

## Results

**Fig. 2:**
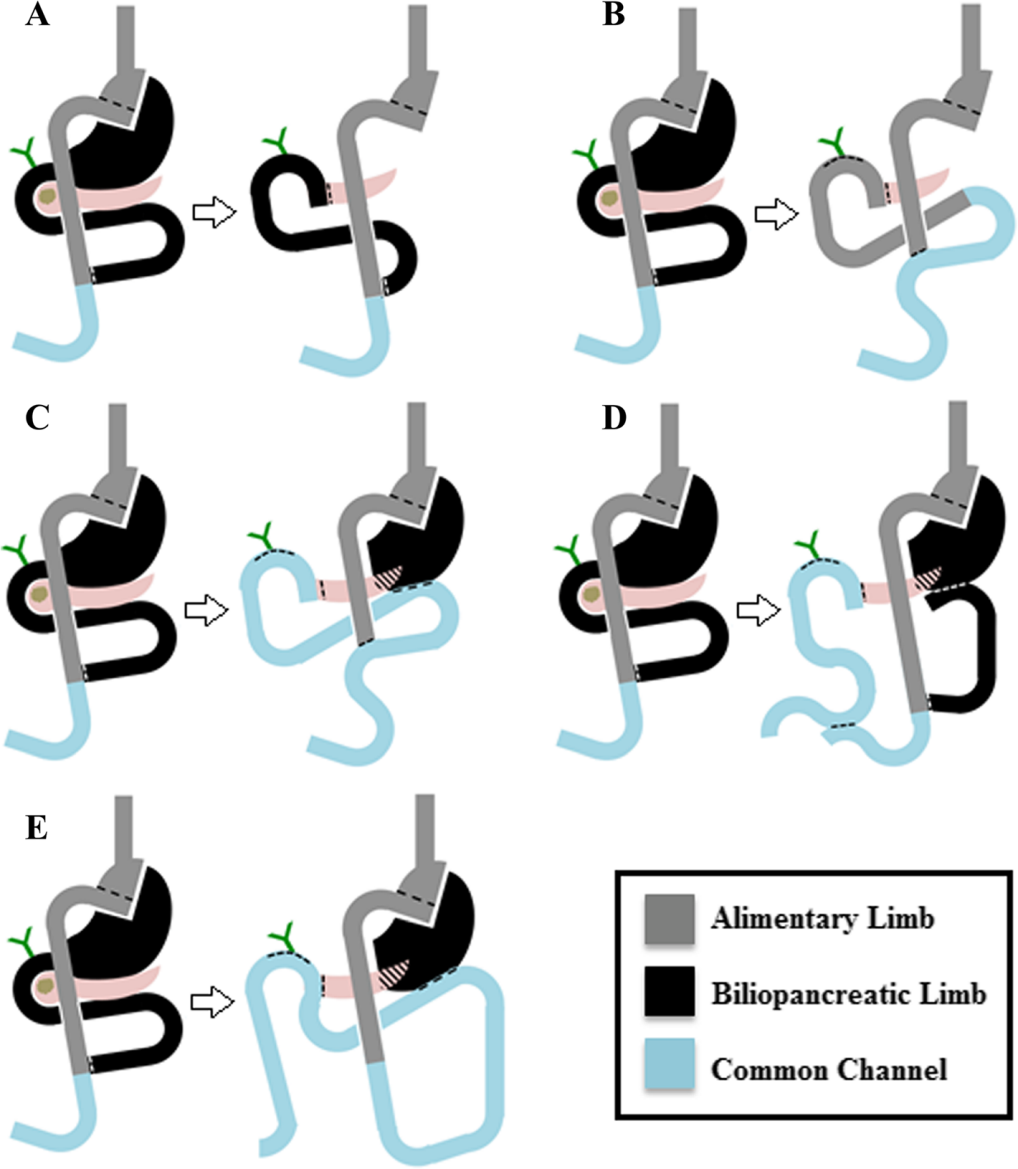
Schematics depicting the different reconstruction options utilized in the literature. Post-RYGB anatomy depicted on left in each figure. **a** Remnant is resected, new biliopancreatic drainage accomplished with distal portion of old biliopancreatic limb. **b** Remnant is resected, new biliopancreatic drainage accomplished with distal portion of old alimentary limb. **c** Remnant is spared, new biliopancreatic drainage and gastric remnant drainage into new limb raised from old common channel, as in our patient. **d** Remnant is spared, new biliopancreatic drainage accomplished with new limb raised from old common channel and gastric remnant is drained into distal portion of old biliopancreatic limb. **e** Remnant is spared, new biliopancreatic and gastric remnant drainage is performed in series and in continuity with old common channel distal to the old jejunojejunostomy

Our search returned 55 English language articles. Eight of these articles were found to specifically address PD after RYGB. In the included articles, 25 patient cases were reported; our institution included an additional case, for a total of 26 cases (Fig. 1) [13–20]. Table 1 contains a synopsis of all reported cases with regard to clinicopathological characteristics. Tables 2 and 3 summarize patient pre-operative, operative, and post-operative characteristics, respectively. Briefly, the patients were predominantly female, in the sixth decade of life (median age 54 years, IQR 52–61). Median interval between gastric bypass and PD was 5 years (IQR 2–11). Patients initially presented with abdominal/back pain [18], jaundice [14], weight loss [9], nausea/vomiting [5], as an incidental finding [4], diarrhea [2], and fever/chills [1]. Computed tomography (CT) was the diagnostic modality of choice in all patients. Pathological diagnoses included pancreatic adenocarcinoma [15], neuroendocrine tumors [3], chronic pancreatitis [3], bile duct fibrosis [2], intraductal papillary mucinous neoplasm [1], duodenal adenocarcinoma [1], and ampullary adenocarcinoma [1]. Procedures included pancreaticoduodenectomy [21], pylorus-preserving pancreaticoduodenectomy [1], and total pancreatectomy [1]. Report of resection margin status [13] and histologic lymph node examination [4] for malignancies were infrequently provided. The gastric remnant was resected in majority of patients [18]. Few surgeons resected the entire biliopancreatic limb [3]. Reconstruction of biliopancreatic drainage was achieved by using distal jejunal segment of the old biliopancreatic limb [22], a new limb raised from the old common channel [3], a new limb raised from the old alimentary limb [1], and creation of a hepaticojejunostomy and pancreaticojejunostomy in-continuity with the old common channel and gastric pouch [1] (Fig. 2). In the reported cases, margin status was reported in 65% of patients with a diagnosis of malignancy. Three post-operative complications were reported: two pancreatic fistulas, one enterocutaneous fistula, and one bile leak from gastrojejunostomy anastomotic breakdown. Of the 17 cases reporting follow-up data (median 27 months), 10 patients had no evidence of disease at last follow-up, 8 died of malignancy.

**Table 1:** Overview of all relevant clinicopathological characteristics in the 26 cases reviewed of post-RYGB patients who underwent pancreaticoduodenectomy

Author	Year	Sex	Age	Diagnosis	Imaging/diagnostic modalities	Years from RYGB	RYGB type	Presenting complaint(s)	Resection specimen	Gastric remnant	Biliary drainage	Pancreatic drainage	Gastric remnant drainage	Feeding access	OR time (min)	EBL (cc)	N staging	Margins	Complications	Oncological Outcome	Follow-up (moths)
Helmick	2010	M	71	IPMN	CT	4	NR	Pain	SPDS	Spared	BL	BL	BL	GT	NR	NR	NR	NR	Bile leak	NED	NR
	2010	F	58	CP	CT	5	NR	Pain, jaundice	SPDS	Spared	BL	BL	BL	GT	NR	NR	NR	N/A	NR	N/A	NR
Swain	2010	F	50	PDAC	CT	1.75	Open	Jaundice, weight loss	SPDS, GR	Resected	BL	BL	N/A	NR	NR	NR	NR	NR	NR	NED	12
		F	55	NET	CT	2	Lap	Incidental finding	SPDS, GR	Resected	BL	BL	N/A	NR	NR	NR	NR	NR	Pancreatic leak	NED	12
		F	61	PDAC	CT	25	Open loop	Incidental finding	SPDS, GR	Resected	BL	BL	N/A	NR	NR	NR	NR	NR	NR	NED	36
		F	56	Ampullary Ca	CT	0.75	Lap	Fever/chills, jaundice	SPDS, GR, BL	Resected	AL	AL	N/A	NR	NR	NR	NR	NR	NR	NED	84
		M	51	NET	CT, Perc	10	Open	Pain	PPPDS, BL	Spared	CC	CC	CC	NR	NR	NR	NR	NR	ECF	NED	NR
Cruz-Muñoz	2011	M	61	NET	CT	0.18	Lap	Incidental finding	SPDS, GR	Resected	BL	BL	N/A	NR	410	250	0	Neg	Pancreatic leak	NED	NR
Khithani	2009	F	60	PDAC	CT	NR	NR	Jaundice, pain	SPDS, GR	Resected	BL	BL	N/A	JT	NR	NR	NR	NR	NR	NED	NR
	2009	F	57	PDAC	CT	NR	NR	Incidental finding	SPDS, GR	Resected	BL	BL	N/A	JT	NR	NR	NR	NR	NR	NED	NR
Rutkoski	2008	F	49	PDAC	CT, US, MRCP	5	Lap	Pain, nausea/vomiting, jaundice	SPDS	Spared	CC	CC	BL	NR	NR	NR	1	Neg	NR	DOD	9
Theodoropoulos	2012	F	53	PDAC	CT	14	Open	Pain	SPDS, BL	Spared	CC distal to J-J	CC distal to J-J	CC distal to J-J	NR	NR	NR	1	Neg	NR	NED	NR
Nikfarjam	2009	F	46	FDBDF	CT, PTC	3	Lap	Jaundice	SPDS, GR	Resected	BL	BL	N/A	NR	480	100	N/A	N/A	NR	N/A	12
	2009	F	72	FDBDF	CT, PTC	5	Open	Jaundice, weight loss	SPDS, GR	Resected	BL	BL	N/A	NR	300	950	N/A	N/A	NR	N/A	12
Peng	2018			PDAC	CT, Perc		Open		SPDS	Spared	AL	AL	CC	NR			NR	Pos	NR	DOD	81
				PDAC	CT		Open		SPDS, GR	Resected	BL	BL	N/A	NR			NR	Pos	NR	DOD	50
				IPMN	CT, PTC		Lap		SPDS, GR	Resected	BL	BL	N/A	NR			NR	Neg	NR	DOD	18
				PDAC	CT		NR		SPDS, GR	Resected	BL	BL	N/A	NR			NR	Pos	NR	DOD	37
				PDAC	CT, PTC		Open		SPDS, GR	Resected	BL	BL	N/A	NR			NR	Pos	NR	AWD	53
				CP	CT, PTC		Open		SPDS, GR, GJ	Resected	BL	BL	N/A	NR			N/A	N/A	NR	N/A	NR
				Duodenal Ca	CT, PTC, Endo		Open		SPDS	Spared	BL	BL	BL	NR			NR	Neg	NR	AWD	57
				PDAC	CT, Perc		Lap		SPDS, GR	Resected	BL	BL	N/A	NR			NR	Neg	NR	AWD	34
				CP	CT, PTC, Endo		Open		SPDS, GR	Resected	BL	BL	N/A	NR			N/A	N/A	NR	N/A	NR
				PDAC	CT, PTC		Open		SPDS, GR, Spleen	Resected	BL	BL	N/A	NR			NR	Neg	NR	DOD	16
				PDAC	CT, PTC		Lap		Pancreas, GR	Resected	BL	BL	N/A	NR			NR	Neg	NR	DOD	27
	Mean		64			10									361	500					
Current report	2015	F	63	PDAC	CT	12	Open	Pain, nausea/vomiting	SPDS, BL	Spared	CC	CC	CC	GT	765	400	2	Neg	Pancreatic fistula	DOD	23

*NR* not reported, *N/A* not applicable, *RYGB* Roux-en-Y Gastric Bypass, *PDAC* pancreatic ductal adenocarcinoma, *NET* neuroendocrine tumor, *FDBDF* focal distal bile duct fibrosis, *CP* chronic pancreatitis, *CT* computed tomography, *US* ultrasound, *MRCP* magnetic resonance cholangiopancreatography, *PTC* percutaneous transhepatic cholangiography, *Perc* percutaneous biopsy, *Endo* endoscopic biopsy, *Lap* laparoscopic, *NR* not reported, *SPDS* standard pancreaticoduodenectomy specimen (pancreatic head, duodenum, antrum, common bile duct, and gallbladder, if present), *PPPDS* pylorus-preserving pancreaticoduodenectomy specimen (pancreatic head, distal duodenum, common bile duct), *GR* gastric remnant, *BL* biliopancreatic limb, *CC* common channel, *AL* alimentary limb, *jej-jej* jejunojejunostomy, *JT* feeding jejunostomy tube placed, *GT* feeding gastrostomy tube, *ECF* enterocutaneous fistula, *NED* no evidence of disease, *DOD* dead of disease, *AWD* alive with disease

**Table 2:** Patient, diagnostic, and pathologic characteristics of post-RYGB patients undergoing pancreaticoduodenectomy in reviewed cases (*N* = 26)

Parameter	Proportion or median	Percentage (%) or IQR
Patient demographics		
Sex (female)	12/15	80
Age (years)	54	52–61
Years from RYGB*	5	2–10
Presenting complaint**		
Pain	18/26	69.2
Jaundice	14/26	53.8
Weight loss	9/26	34.6
Nausea/vomiting	5/26	19.2
Incidental finding	4/26	15.4
Diarrhea	2/26	7.7
Fever/chills	1/26	3.9
Preoperative diagnostic modality**		
CT	26/26	100
PTC	9/26	34.6
Percutaneous biopsy	3/26	11.5
Endoscopic biopsy	2/26	7.7
US	1/26	3.9
MRCP	1/26	3.9
Pathologic diagnosis		
PDAC	14/26	53.8
NET	3/26	11.5
CP	3/26	11.5
FDBDF	2/26	7.7
IPMN	1/26	3.9
Duodenal Ca	1/26	3.9
Ampullary Ca	1/26	3.9

*IQR* interquartile range, *RYGB* Roux-en-Y Gastric Bypass, *PDAC* pancreatic ductal adenocarcinoma, *NET* neuroendocrine tumor, *FDBDF* focal distal bile duct fibrosis, *CP* chronic pancreatitis, *IPMN* intraductal papillary mucinous neoplasm, *CT* computed tomography, *US* ultrasound, *MRCP* magnetic resonance cholangiopancreatography, *PTC* percutaneous transhepatic cholangiography

*Only 11 cases with reported RYGB details

**Possible for one patient to have multiple presenting symptoms or diagnostic modalities

**Table 3:** Operative and post-operative characteristics of post-RYGB patients undergoing pancreaticoduodenectomy in reviewed cases (*N* = 26)

Parameter	Proportion	Percentage (%)
Pancreatic resection performed		
Pancreaticoduodenectomy	24/26	92.3
Pylorus-preserving pancreaticoduodenectomy	1/26	3.8
Total pancreatectomy	1/26	3.8
Resection specimen (in addition to standard PD specimen)		
Gastric remnant	18/26	69.2
Old biliopancreatic limb	3/26	11.5
Reconstruction of biliopancreatic drainage		
Biliopancreatic limb	21/26	73.1
New limb from common channel	3/26	19.2
New limb from alimentary limb	1/26	3.8
Common channel (limb in continuity)	1/26	3.8
Drainage of gastric remnant		
Biliopancreatic limb	4/8	50
New limb from common channel	3/8	37.5
Common channel (limb in continuity)	1/8	12.5
Enteral feeding access		
Gastrostomy tube	3/26	11.5
Jejunostomy tube	2/26	7.7
Oncologic outcome		
NED	10/21	84.6
DOD	8/21	7.7
AWD	3/21	7.7
Median follow-up (months)	17/26	27

*PD* pancreaticoduodenectomy, *IQR* interquartile range, *NED* no evidence of disease, *DOD* dead of disease, *AWD* alive with disease

## Discussion

Obesity is a known risk factor for pancreatic cancer [1, 2]. As in our patient, the diagnosis of a resectable pancreatic head mass requires a PD, classically involving en bloc resection of the pancreatic head, distal stomach and duodenum, common bile duct, and gallbladder. Reconstruction is typically achieved by creation of a pancreaticojejunostomy, hepaticojejunostomy, and gastrojejunostomy, in series. However, given the anatomical alterations, post-PD reconstruction requires greater forethought in the post-RYGB population. Although infrequently reported, these procedures can be longer in duration with a greater potential for morbidity. All potential reconstruction options found in the literature are summarized in Fig. 2. Patient selection and preoperative planning to identify resectable disease are paramount [22].

Classically, the RYGB reconstruction involves creating an anastomosis of the jejunal alimentary limb to the gastric pouch, which is connected to a separate biliopancreatic limb. This reconstructed anatomy produces both restrictive and malabsorptive components for weight loss. A subsequent PD requires reconstruction of biliary and pancreatic drainage which had previously been achieved by the biliopancreatic limb. If there remains sufficient length on this limb, most authors recommend using the distal jejunal segment of this limb to accomplish drainage [12]. In certain cases, the entire biliopancreatic limb may need to be resected, requiring construction of a new limb. The source of this new biliopancreatic limb may arise from the old common channel distal to the jejunojejunostomy (Fig. 2a) or from the amputated alimentary limb, the distal part of which becomes utilized for a hepaticojejunostomy and pancreaticojejunostomy (Fig. 2b). In both circumstances, a separate jejunojejunal anastomosis will need to occur.

If the gastric remnant is not resected, drainage of this channel must be factored into the reconstruction. The more commonly reported reconstruction consists of using a new limb from the old common channel for hepaticojejunostomy, pancreaticojejunostomy, and remnant gastrojejunostomy (Fig. 2c). Our patient underwent a PD with resection of the old biliopancreatic limb, sparing the gastric remnant and placement of a feeding gastrostomy tube (pre-operative albumin 2.6 g/dL). We recreated the biliopancreatic limb from the prior common channel. Since the gastric remnant was left in situ, we performed a jejuno-gastric remnant anastomosis in series with this same limb. Alternatively, another author reported using the old biliopancreatic limb for the remnant gastrojejunostomy while raising a new limb from the old common channel for the hepaticojejunostomy and pancreaticojejunostomy (Fig. 2d) [19]. While physiologically appropriate, this does increase the number of anastomoses and possibility of morbidity. Of interest, one author reported construction of a hepaticojejunostomy and pancreaticojejunostomy with the common channel far distal to the jejunojejunostomy and in continuity with the gastric pouch and alimentary limb (Fig. 2e) [20]. This is inadvisable, as there is risk for reflux of enteric contents into the biliary tree, increasing the incidence of cholangitis [12]. Each of these reconstruction methods reflects surgeon preference, as well as anatomic considerations posed by individual patients.

Though less commonly performed in the reviewed cases (30.8%), there may be advantages to retaining the gastric remnant during PD in the post-RYGB patient including retained physiologic function, nutritional support, and ease of future diagnostic and therapeutic interventions. Since the first gastric bypass reported by Ito and Mason in 1967, interest in gastrointestinal tract physiologic changes brought on by altered surgical anatomy persists [23]. Particular focus was paid to the importance of the excluded stomach (remnant) for motility and secretory function, plus the impact of surgical discontinuity of the gastric pouch. Printen et al. and Mason et al. published studies in which pre- and post-operative gastric pH and secretions were found to be identical [21, 24]. Both studies pointed to retention of vagal innervation to the gastric remnant. Given that motor migratory complex (MMC) initiates mainly from the interstitial cells of Cajal at the gastric antrum, peristalsis remains present in the gastric remnant. The propulsion of gastric, biliary, and pancreatic secretions into the common channel after bypass is evidence of this [25].

The gastric remnant also retains importance in the body’s endocrine and exocrine functions in the post-RYGB anatomy. The increase in levels of incretin hormones in the post-RYGB, such as glucagon-like peptide-1 (GLP—1) and gastric inhibitory peptide (GIP), is well studied [26, 27]. Severe hypogylcemia after RYGB is an increasingly recognized complication, possibly due to hyperinsulinemia and β-cell proliferation from increased GLP-1 activity, or failure of islet cell regression in diabetic patients post-RYGB [28]. McLaughlin et al. successfully treated medically refractory hypoglycemia after RYGB with enteral feeds through a gastrostomy tube placed in the gastric remnant. This corrected post-surgical derangements in glucose, insulin, GLP-1, glucagon, and GIP after oral food intake [29].

Additionally, 16% of partial gastrectomy patients develop B12 deficiency and this rate increases with elapsed-time post-surgery, with some patients presenting with B12 deficiency 10 years or more after partial gastrectomy [30, 31]. This effect is, in part, due to both the restrictive and malabsorptive aspects of the procedure. The early satiety induced by gastric restriction leads to reduction in hydrochloric acid and pepsin production. Reduction of available B12 from food and decreased exposure to intrinsic factor (IF) producing cells subsequently leads to B12 malabsorption [32]. B12 deficiency can have profound implications on overall health, anemia, and neurologic disorders [33]. Recently, Sala et al. demonstrated that post-RYGB risk of B12 deficiency may also be the result of changes in upregulation of B12 pathway-encoding genes [32]. The gastric remnant may also be capable of reasserting its function for intrinsic factor production, as well as modulating secondary measures of intestinal B12 absorption via increased production of transcobolamin II, which binds B12 after its release from IF in the ileum [32].

Delayed gastric emptying (DGE) is a common post-operative complication of PD, occurring in 15–40% of patients [34, 35]. Suspected causes of DGE include removal of the motilin-secreting duodenum, gastric irritation from bile, and interruption of the myoneural pathways in the bowel wall [36]. Furthermore, DGE has been associated with deep space infection and leak, although causal relationships remain poorly defined [37]. Regardless of etiology, DGE interferes with resumption of normal diet and post-operative nutrition. In the post-RYGB patient, this complication may be amplified due to the altered physiology. A study by Dutra et al. on Wistar rats previously showed that increased length of the biliopancreatic limb could serve as a functional barrier to gastric emptying while offering no advantages in preventing enterogastric reflux [38]. Gustavsson et al. analyzed outcomes of 234 patients who underwent total or subtotal gastrectomy with Roux-en-Y reconstruction, demonstrating that those with gastric dysmotility had longer roux-limbs (mean 41 cm), and shortening these limbs improved symptoms [39]. Additional studies of dysmotility after RYGB focus on reconstruction of intestinal anatomy and disruption of MMCs, displacement of the native pacemaker cells of the gut by slower ectopic signals, and changes in the metabolic and endocrine regulation of these events [40]. Preservation of the gastric remnant with subsequent reconstruction in this patient population may allow for improved physiologic parameters, and diagnostic and therapeutic interventions.

Intolerance to oral intake is multi-factorial in the PD-RYGB population and can sufficiently compromise patient nutrition, requiring further intervention for enteral supplementation. An intact gastric remnant provides the opportunity to leave a remnant gastrostomy tube for post-operative decompression and enteral nutrition post-PD [41]. In our patient, we placed a feeding gastrostomy tube in the gastric remnant, as malnutrition is associated with adverse outcomes following PD, especially in a patient who has already had significant weight loss and hypoalbuminemia [42, 43]. These complications include sepsis, impaired wound healing, and pancreatic fistula formation [44–46]. A recent systematic analysis of different enteral routes of nutrition (15 articles, 3474 patients) found that gastrojejunostomy feeding was associated with the shortest hospital stay (mean 15 days) and lowest incidence of delayed gastric emptying (6%) [47]. Barbour et al. published an experience with five patients requiring pancreatic resections after RYGB in which a gastrostomy tube in the gastric remnant was successfully used for decompression and, later, to supplement with enteral nutrition [41]. This may be particularly significant in the post-RYGB patient as hypoalbuminemia (< 3.5 mg/dL) may occur in up to 13% [48]. Remnant gastrojejunostomy placement may help overcome these issues.

Preservation of the gastric remnant also aids in post-PD diagnostic and therapeutic intervention. Performing ERCP in the altered anatomy post-PD can be technically challenging even with an intact stomach. Many endoscopists favor anterograde EUS access for pancreatic duct interventions post-PD. Such interventions may be difficult, from a small gastric pouch. Chalal et al. demonstrated only a 51% success rate in 88 ERCPs performed in post-PD patients at the Mayo Clinic (2002–2005) [49]. Laparoscopic-assisted ERCP to access the remnant stomach, as opposed to the jejunum or gastric pouch, may provide a number of advantages, especially in terms of supporting access in a position similar to native anatomy [50]. Several investigators described success with EUS access of the remnant stomach to allow for laparoscopic-assisted ERCP through the remnant in post-Roux-en-Y anatomy [51].

While reversal of RYGB is an uncommon procedure, the related literature demonstrates improvement in post-operative morbidities related to the post-RYGB anatomy, lending support to the concept that the gastric remnant retains much of its physiologic function [52, 53]. In cases in which the post-RYGB patient develops severe complications (acute hypoglycemia, weight regain, intractable diarrhea, extreme dumping syndrome, cachexia), reversal of the bypass to normal anatomy commonly leads to resolution of symptoms [52]. Vilallonga et al. published an experience with 20 patients in which they describe laparoscopic reversal of RYGB with resolution of most complications, although few patients did develop gastroesophageal reflux disease (three patients) and diarrhea (one patient), secondary to damage of the vagus nerves [54]. Similarly, Pernar et al. showed resolution of the predominant symptoms in 15 of 19 patients, including 6 weaned from total parenteral nutrition (TPN). Given that the gastric remnant retains its function, this procedure remains an option, though it should only be considered in select patients.

Despite the benefits of leaving the gastric remnant in situ, there may be technical advantages to resecting the gastric remnant en bloc with the specimen [55–57]. First, it obviates the need for a jejuno-gastric remnant anastomosis and associated potential complications. However, an important consideration, a prospectively collected, multicenter study by Smith et al. of nearly 4500 gastric bypass patients, found only 1% clinically significant gastrojejunostomy leak rate [57]. In turn, other recent series report anastomotic stricture rates between 4.8 and 7.3% [55, 56]. Therefore, by resecting the remnant and avoiding additional anastomoses, the surgeon may simplify the subsequent reconstruction while avoiding potential morbidity. In contrast, preserving the gastric remnant may also allow for future development of bleeding, ulceration, or undetected malignancy, although the overall risk of developing gastric and esophageal malignancies is reportedly rare in the post-RYGB patient [58–60].

This systematic review has limitations. Few publications exist interrogating this particular area; therefore, there remains a paucity of data with which to develop clear, evidence-based guidelines for reconstruction options. Indeed, with both the increasing incidence of pancreatic cancer and number of patients undergoing bariatric procedures, this clinical scenario will become more prevalent, making discussions of the surgical options more frequent and relevant. Furthermore, gastric remnant preservation, while currently performed less frequently, may be advantageous from a multidisciplinary standpoint, but clearly requires further investigation [61]. Although not discussed, decisions regarding preoperative diagnostic modalities or perioperative neoadjuvant and adjuvant treatments for this unique, but expanding, patient population deserves further review.

## Conclusions

Pancreaticoduodenectomy after Roux-en-Y gastric bypass is a complex procedure that is rarely performed. Varying practice patterns reflect the complexity of the surgery and diversity of surgeon preference. Few publications exist to develop recommendations, yet there is a growing need to provide evidence for the safest and most effective method of resection and reconstruction in this growing population. Regardless of reconstruction used, the most important goal should be definitive resection (R0), followed by consideration for the patient’s future quality of life and further treatment.

## Abbreviations

CT: Computed tomography
DGE: Delayed gastric emptying
ERCP: Endoscope retrograde cholangiopancreatography
IQR: Inter-quartile range
MMC: Motor migratory complex
PD: Pancreaticoduodenectomy
RYGB: Roux-en-Y gastric bypass
TPN: Total parenteral nutrition
